# Nutzen der partizipatorischen Mitwirkung von PatientInnen an der Entwicklung einer dermatologischen Therapie-App – ein Bericht aus der Praxis

**DOI:** 10.1007/s00105-024-05326-7

**Published:** 2024-03-22

**Authors:** Anne Koopmann, Anna Maria Pfeifer, Lara Schweickart, Nathalie Biniaminov, Valentin Haas, Philipp Marquardt, Astrid Gößwein, Christopher Czaban, Sergey Biniaminov, Mara Blauth, Caroline Glatzel, Christoph Zimmermann, Wilhelm Stork, Victor Olsavszky, Astrid Schmieder

**Affiliations:** 1 Klinik für Abhängiges Verhalten und Suchtmedizin, Zentralinstitut für Seelische Gesundheit Mannheim, Universität Heidelberg, Mannheim, Deutschland; 2 Feuerlein Centrum für Translationale Suchtmedizin (FCTS), Universität Heidelberg, Heidelberg, Deutschland; 3 FZI Forschungszentrum Informatik, Karlsruhe, Deutschland; 4 HS Analysis GmbH, Haid-Und-Neu-Str. 7, 76131 Karlsruhe, Deutschland; 5 DataSpark GmbH, Frankfurt, Deutschland; 6 Karlsruher Institut für Technologie, Karlsruhe, Deutschland; 7 Klinik für Dermatologie, Venerologie und Allergologie, Universitätsmedizin Mannheim, Universität Heidelberg, Mannheim, Deutschland; 8 Klinik für Dermatologie und Venerologie, Universitätsklinikum Würzburg, Haus D8, Josef-Schneider-Str. 2, 97080 Würzburg, Deutschland

**Keywords:** Patientenzentrierte Versorgung, Psychologischer Stress, Psoriasis, Maschinelles Lernen, Künstliche Intelligenz, Patient-centered care, Stress, psychological, Psoriasis, Machine learning, Artificial intelligence

## Abstract

Etwa 2 % der Bevölkerung in Deutschland leiden an Psoriasis. HybridVITA hat eine mobile Applikation (App) entwickelt, mit der Psoriasispatienten ihren Krankheitsverlauf und ihre aktuelle psychische Belastung eigenständig zu Hause dokumentieren und an ihre Behandler übermitteln können. Die HybridVITA-App wurde in enger Zusammenarbeit mit den Nutzergruppen entwickelt, um sie optimal an deren Bedürfnisse anzupassen. Zwei interaktive Workshops mit den Nutzergruppen und den technischen Entwicklern der App waren ein Kernelement dieses Prozesses. Die Workshops dienten der Ermittlung von Bedürfnissen und Verbesserungsvorschlägen der verschiedenen Nutzergruppen sowie der Formulierung von „user stories“ für die Weiterentwicklung der App mithilfe der Scrum-Methode. Durch den partizipatorischen Ansatz des Workshops konnte das Projektteam wertvolles Praxiswissen bereits in einem frühen Entwicklungsstadium sammeln. Das Wissen um mögliche Hürden in einer frühen Projektphase ermöglichte es dem Team, Lösungen für Probleme zu finden, bevor die App in der dermatologischen Versorgung eingesetzt wird. Aus unserer Sicht kann eine partizipative und patientenzentrierte Herangehensweise bei der Entwicklung von Gesundheits-Apps einen großen Erkenntnisgewinn für die Entwickler bedeuten.

## Abstract (en)

Approximately 2% of the German population suffer from psoriasis. HybridVITA has developed a mobile application (app) that enables psoriasis patients to independently document the progression of the disease and the current psychological stress at home. The HybridVITA app was created in close collaboration with user groups to ensure optimal adaptation to their needs. Two interactive workshops were held with the user groups and the technical developers of the app as a core element of the developmental process. The workshops identified the needs and suggestions for improvement of the various user groups and formulated user stories for the further development of the app using the Scrum method. The participatory approach of the workshop enabled the project team to gather valuable practical knowledge at an early stage of development. The team’s awareness of potential obstacles during the early stages of the project enabled them to proactively identify and address these issues prior to implementing the app in dermatological care. We are confident that a patient-centered and participatory approach to health app development can provide valuable insights for developers.

Etwa 2 % der Bevölkerung in Deutschland sind von Psoriasis betroffen mit teilweise gravierenden Auswirkungen auf ihre Lebensqualität [3]. Obwohl frühere Studien [1] zeigten, dass Psoriasispatienten von einer engen interdisziplinären Zusammenarbeit von Dermatologen und Rheumatologen profitieren würden, stellt das interdisziplinäre therapeutische Management für alle beteiligten medizinischen Disziplinen eine große Herausforderung dar – mit konsekutiv verzögerten Behandlungsabläufen, erhöhten Hospitalisierungsraten und Einschränkungen der physischen Funktionalität, psychischen Beeinträchtigungen und Morbidität für die Betroffenen [4].

Ziel des Projektes HybridVITA (Medizinische Betreuung von Patienten mit chronischen Hauterkrankungen durch eine App-basierte *Hybrid*lösung mit kontaktloser *VI*so-*TA*ktiler Diagnostik) ist die Entwicklung einer mobilen Applikation (App), mit der Psoriasispatienten auf der Basis von künstlicher Intelligenz (KI) und maschinellem Lernen (ML) ihren Krankheitsverlauf und ihre aktuelle psychische Belastung selbstständig zu Hause dokumentieren und an ihre Behandler übermitteln können. So können Therapieregimes ohne zeitlichen Verzug angepasst werden, wodurch Schübe sowie weitere psoriasisassoziierte Erkrankungen und konkomitante psychische Symptome frühzeitig erkannt und behandelt werden können. Um dies zu ermöglichen, erstellen die Patienten über die HybridVITA-App einmal wöchentlich Aufnahmen betroffener Hautstellen zur KI- und ML-basierten Analyse und füllen Fragebögen zu ihrem aktuellen Befinden aus. Bei einer Verschlechterung der Psoriasissymptomatik und/oder der psychischen Beschwerden mit Überschreitung definierter Schwellenwerte generiert die App Warnhinweise und Empfehlungen zum weiteren Vorgehen für die Patienten und bietet eine kurzfristige ärztliche Videovisite an. Dies ermöglicht eine unkomplizierte Kontaktaufnahme zu den Behandlern auch ohne einen persönlichen Praxis‑/Ambulanzbesuch und ein niederschwelliges psychotherapeutisches Unterstützungsangebot. Darüber hinaus haben Betroffene die Möglichkeit, an Online-Patientenschulungen zu dermatologischen Themen sowie zu den Themen Depression, Angst und Stressbewältigung im Alltag teilzunehmen.

Um die HybridVITA-App entsprechend den Bedürfnissen der Nutzergruppen (niedergelassene und klinisch tätige Dermatologen sowie Psoriasispatienten) zu gestalten, werden diese in einem partizipativen Ansatz in den Entwicklungsprozess eingebunden. Ein Kernelement dieses Prozesses stellten 2 interaktive Präsenz-Workshops mit den Nutzergruppen und den technischen Entwicklern der App dar. Diese fanden im November und Dezember 2022 am Zentralinstitut für Seelische Gesundheit Mannheim und dem Universitätsklinikum Würzburg statt. Mit an Psoriasis erkrankten Patienten und ihren Angehörigen, klinisch bzw. im niedergelassenen Bereich tätigen Dermatologen sowie Psychiatern und Software-Entwicklern waren sämtliche am Entwicklungsprozess der HybridVITA-App beteiligten Gruppen vertreten.

## Ablauf der Workshops

Nach einer thematischen Einführung in die telemedizinischen Behandlungsmöglichkeiten chronischer Hauterkrankungen und in die Ziele des Projekts HybridVITA wurden interdisziplinäre Austauschforen durchgeführt. Hierzu wurden Gruppen mit mindestens einem Vertreter aus allen späteren Nutzergruppen der App sowie einem Moderator aus dem Projektteam gebildet. So konnten die Probleme in der Zusammenarbeit während der Behandlung chronischer Hauterkrankungen aus Sicht der einzelnen Stakeholder identifiziert und Lösungsansätze erarbeitet werden. Das Ergebnis der Gruppendiskussion wurde in einem 15-minütigen Plenum mit allen Workshop-Teilnehmern diskutiert.

Anschließend wurden das Design und die Inhalte der geplanten HybridVITA-App in einem interaktiven Vortrag vorgestellt. Hierbei bestand für die Nutzer die Möglichkeit, direkt Feedback zur optischen Gestaltung und Funktionalität der Benutzeroberflächen für die einzelnen Gruppen zu geben, Fragen zu stellen, eigene Vorschläge zur grafischen oder inhaltlichen Gestaltung einzubringen und diese sowohl untereinander als auch mit den IT-Experten des Entwicklungsteams zu diskutieren.

Basierend auf den so gewonnenen Erkenntnissen über die Bedürfnisse und Verbesserungsvorschläge der verschiedenen Nutzergruppen konnten mithilfe der Scrum-Methode (s. unten) „user stories“ für die Weiterentwicklung der Anwendung durch IT-Experten formuliert werden [2].

### Scrum-Methode

Die Scrum-Methode ist ein agiles Framework, das multidisziplinäre Entwicklungsteams bei der Priorisierung von Aufgaben unterstützt. Die Arbeitsaufträge für die Entwicklungsteams werden in der Regel aus der Sicht der späteren Nutzer in Form von „user stories“ formuliert. „User stories“ stellen somit Elemente dar, über die Nutzer die Entwicklung eines Systems und letzten Endes dessen Funktionalität beeinflussen können.

## Ergebnisse der Workshops

Dermatologen wünschen sich von der HybridVITA-App eine Vereinfachung ihrer Arbeitsabläufe sowie eine möglichst zeit- und kostensparende Implementierung der Anwendung im Klinik- bzw. Praxisalltag. Auch eine Zeitersparnis bei der Betreuung und Behandlung von Patienten durch das Nutzen der App ist ein häufiger Wunsch seitens der Dermatologen. Zeitliche Effizienz bei der Therapie der Psoriasis und ihrer Begleiterkrankungen war auch für die teilnehmenden Patienten ein wichtiges Anliegen. Hierzu gehörten für die Patienten auch eine möglichst umfassende Integration aller behandlungsrelevanten Aspekte mithilfe der App als zentral genutztes Tool und die Zugänglichkeit der gesammelten Daten für andere Fachärzte.

Weiterhin wünschten sich die Patienten ein angemessenes Konzept zur Beurteilung von psoriasisassoziierten Schmerzen. Hierfür ist in der App eine KI-basierte Beurteilung vorgesehen, in der ein standardisierter Text vorgelesen werden soll und Schmerzen anhand der Mimik der Patienten bewertet werden sollen. Da im medizinischen Bereich fortlaufend nach Tools zur objektivierbaren Schmerzbeurteilung gesucht wird, wurde neben der sich durch ihre Subjektivität auszeichnenden visuellen Analogskala ein KI-gestützter – und damit objektiver – Ansatz für die HybridVITA-App ausgewählt. In diesem Zusammenhang wurde von einigen Patienten allerdings die Sorge geäußert, dass Schmerzen anhand der Mimik KI-abhängig als zu gering eingeschätzt werden könnten und ihren subjektiven Angaben zur Schmerzintensität so zu wenig Beachtung geschenkt werden könnte. Es ist daher der Einsatz sowohl der KI-basierten Schmerzbeurteilung als auch der visuellen Analogskala geplant, was zudem auch einen Vergleich beider Ansätze in der Praxis ermöglicht.

Die ärztlichen Teilnehmer äußerten die Sorge, dass aufgrund der noch nicht hinreichenden Etablierung telemedizinischer Anwendungen insbesondere im niedergelassenen Bereich eine angemessene Vergütung bei Nutzung der App möglicherweise nicht gewährleistet wäre.

## Diskussion

Durch den partizipativen Ansatz des Workshops konnten in einem frühen Stadium der App-Entwicklung wertvolle Erkenntnisse aus der Praxis gewonnen werden. Wünsche und Erwartungen an die HybridVITA-App, aber auch Bedenken konnten herausgearbeitet und gesammelt werden. Dies erleichtert eine optimale Anpassung der App an die Bedürfnisse der einzelnen Nutzergruppen hinsichtlich des Designs und der Funktionalität. Der Austausch zwischen den verschiedenen Nutzergruppen war hierbei besonders wertvoll. Allerdings können insbesondere Fragen nach Vergütung sowie Implementierbarkeit der App im Klinik- bzw. Praxisalltag zum gegenwärtigen Zeitpunkt noch nicht abschließend beantwortet werden und bedürfen einer weiteren Klärung.

Die Organisation des Workshops war mit einigen Herausforderungen verbunden. So gestaltete sich im Vorfeld der Workshops vor allem die Gewinnung von Teilnehmern als schwierig. Bei manchen potenziellen Teilnehmern war die Bereitschaft bzw. Möglichkeit zur Teilnahme am Wochenende begrenzt, weshalb der Workshop an beiden Standorten unter der Woche durchgeführt wurde, was bei anderen angefragten Teilnehmern zu Konflikten mit beruflichen Verpflichtungen führte. Darüber hinaus gab es für die Anwender keinen unmittelbaren Nutzen aus der Teilnahme am Workshop, während gleichzeitig ein erheblicher Zeit- und Arbeitsaufwand entstand.

Hierdurch entstand bereits eine Vorselektion insbesondere der teilnehmenden Patienten, da für den Workshop nur sehr motivierte Teilnehmer gewonnen werden konnten. Dies könnte Effekte auf die Generalisierbarkeit der Patientenbedürfnisse und Anmerkungen zu der App haben.

Darüber hinaus ist es in medizinischen Fachkreisen aufgrund eingeschränkter Expertise in den Bereichen Didaktik und Evaluation oft herausfordernd, methodisch aufwendige und ansprechende Workshops zur partizipativen Einbeziehung von Patienten zu gestalten.

Einige der herausgearbeiteten Probleme, wie etwa die unklare finanzielle Vergütung für Dermatologen bei Verordnung der HybridVITA-App, konnten im Rahmen des Workshops nicht gelöst werden. Die Kenntnis solcher möglichen Hindernisse in einem frühen Projektstadium ermöglicht es dem Projektteam, für diese Probleme vor Beginn des geplanten Einsatzes der App in der dermatologischen Versorgung Lösungen zu finden, um eine möglichst hohe Akzeptanz der HybridVITA-App bei den avisierten Nutzern zu erreichen und so die interdisziplinäre Versorgung von Psoriasispatienten nachhaltig zu verbessern.

## Fazit für die Praxis

- Ein partizipativer, patientenzentrierter Ansatz bei der Entwicklung von Gesundheitsanwendungen kann aus unserer Sicht einen großen Erkenntnisgewinn für die Entwickler beinhalten.
- Vorteile für die Patienten müssen in der kommenden prospektiven klinischen HybridVITA-Studie untersucht werden.
- Durch die Arbeit mit Scrum und anderen interaktiven Methoden konnte im Projekt ein wichtiger, konstruktiver Austausch zwischen den einzelnen Interessengruppen gefördert werden, was sich möglicherweise auch auf andere, ähnliche Projekte anwenden lässt.
- Für Workshops und vergleichbare Formate sollte unserer Erfahrung nach ein großzügig bemessener zeitlicher Rahmen gesetzt werden, um ausreichend Raum für das Erarbeiten und Diskutieren möglichst vieler für die verschiedenen Interessengruppen relevanter Aspekte zu schaffen.
